# Reliability and validity of the six-minute step test in assessing the functional capacity of hemodialysis patients

**DOI:** 10.7717/peerj.19157

**Published:** 2025-07-08

**Authors:** Carla Cristina Lima, Maria Eduarda Godoy, Débora Kellen Ferreira Fratoni, Elaine Paulin

**Affiliations:** Universidade do Estado de Santa Catarina, Florianópolis, Brazil

**Keywords:** Hemodialysis, Functional capacity, Reliability, Validity, Kidney disease

## Abstract

**Purpose:**

Hemodialysis patients often experience functional limitations that are challenging to assess due to equipment and space constraints. The 6-min step test (6MST) requires little space and a small standardized ergometer, making it more accessible and feasible in various healthcare settings, when compared to the cardiopulmonary exercise test (CPET) and the 6-min walk test (6MWT). Thus, the aim of this study is to evaluate the test-retest reliability and concurrent validity of the 6MST for assessing the functional capacity of hemodialysis patients.

**Methods:**

Assessments conducted included anthropometry, spirometry, 6MWT, and 6MST. These two tests were randomized and performed on alternate days. Two 6MSTs and two 6MWTs were conducted with a minimum interval of 30 min between them.

**Results:**

The study involved 32 participants, 67% men (*n* = 22) and 33% women (*n* = 10), with an average age of 57 ± 13 years and body mass index (BMI) of 28.6 ± 5.2 kg/m^2^. The 6MST demonstrated high test-retest reliability (ICC = 0.94 (95% CI [0.85–0.97]; *p* < 0.001)) and a strong correlation between the number of steps performed in the 6MST and the distance covered in the 6MWT (r = 0.87; *p* < 0.001). A learning effect was observed, with a 7% improvement on retesting.

**Conclusion:**

The 6MST provides a feasible, reliable assessment option for hemodialysis patients and could be easily implemented in clinics lacking space and specialized equipment. Limitation of the present study are being a single-center investigation with a small sample size, thus limiting generalizability, and the variability of patient conditions, which may have influenced the results. Future research should explore the 6’ long-term stability, responsiveness, and impact on patient-centered outcomes, as well as its applicability across different hemodialysis populations and clinical settings, to improve its clinical usefulness.

## Introduction

Chronic kidney disease (CKD) leads to permanent and irreversible physiological disturbances. Exacerbation of inflammatory factors and imbalances in muscle metabolism affect the functionality and quality of life of kidney patients (Hovyzian et al., 2017), and may be related to disease complications (Fassbinder et al., 2015), making it necessary to assess functional capacity through functional tests.

In terms of functional capacity, in addition to the effects of CKD itself, the restrictions imposed by dialysis exacerbate patients’ reduced submaximal exercise capacity, limiting daily activities from treatment onset. Moreover, the chronic inflammation present in this population contributes to tissue malnutrition, directly affecting functionality (Oliveira, Vieira & Bündchen, 2018; Barcellos et al., 2018). The impairment of multiple systems combined with symptoms such as fatigue, vascular dysfunction, and functional decline increase the risk of hospitalization (Jhamb et al., 2016).

The maximal cardiopulmonary exercise test (CPET) is considered the gold standard for assessing exercise tolerance. However, it is costly, unavailable in most centers, especially hemodialysis clinics, and requires specialized staff to administer it test and interpret the results (Ritt et al., 2021; Pessoa et al., 2014). An alternative to CPET is the 6-min walk test (6MWT), a widely studied, valid, and reproducible test used in different populations (Pessoa et al., 2014), including those with CKD. Relationships have been identified between reduced functional capacity assessed by the 6MWT and clinical and physiological manifestations of the disease (Ibrahim & Abdulazeez, 2021). However, since the 6MWT is a field test that requires a 30-m space, it is not always a feasible option in different settings for hemodialysis patients.

By contrast, the 6-min step test (6MST) requires little space, and only a small standardized ergometer, making it more accessible and feasible across several healthcare settings. The 6MST has been validated in populations with exercise limitations similar to those imposed by CKD, such as individuals with chronic obstructive pulmonary disease (COPD), interstitial lung disease and heart failure (Pessoa et al., 2014). These conditions exhibit systemic alterations comparable to those found in hemodialysis patients. Additionally, the 6MST shows a good correlation with the 6MWT in hospitalized COPD patients, suggesting that it could potentially replace the 6MWT in hospital settings (Schnaider & Karsten, 2017). However, its reliability and validity have not yet been established in hemodialysis patients, who may have unique functional limitations.

Measurement instruments are fundamental in research, clinical practice, and health assessment, providing evidence-based insights for clinical decisions. Reliability ensures that an instrument, like 6MST, consistently reproduces results across repeated measurements, which is vital for tracking patient progress over time. Concurrent validity, assessed against the 6MWT, ensures that the 6MST accurately measures functional capacity, a key indicator of physical performance. This is particularly important in hemodialysis settings where space and specialized equipment may be limited. Including reliable and valid tools allows practitioners to make better-informed decisions and optimize patient care (de Souza, Alexandre & de Guirardello, 2017).

Thus, due to the limited availability of valid tests for assessing functional capacity, the 6MST emerges as an alternative for patients with CKDs. However, its psychometric properties need further investigation. This study aims to assess the test-retest reliability and concurrent validity of the 6MST in hemodialysis patients, hypothesizing that it will demonstrate high reliability and validity compared to the 6MWT.

## Method

This is a cross-sectional, single-centered study approved by the Human Research Ethics Committee of the Santa Catarina State University (UDESC) (certificate of ethical appreciation presentation: 23430619.4.0000.0118). The study sample consisted of hemodialysis patients of both sexes admitted for hemodialysis treatment. Inclusion criteria for the study were: (1) individuals diagnosed with CKD who had been undergoing regular hemodialysis treatment for at least 6 months; (2) aged between 20 and 75 years; (3) not experiencing worsened conditions and under medical supervision; (4) individuals who did not exhibit uncontrolled hypertension, recent ischemic heart disease (3 months or less), unstable angina, or severe cardiac arrhythmias; (5) absence of diseases that would limit the assessment protocols; (6) individuals who were not engaged in any form of physical training and/or who had not trained in the past 6 months. Exclusion criteria included: (1) inability to perform any of the study assessments (due to lack of understanding or cooperation) and (2) cardiorespiratory instability (intolerant dyspnea, angina, pallor, sweating, syncope) during the tests. All individuals included read and signed the informed consent form.

### Study design

Assessments were conducted over two non-consecutive days, alternating with hemodialysis sessions. On the first day, the following were performed: medical history, anthropometric data collection, and one of the randomly assigned functional tests (6MST or 6MWT). On the second day, the other functional test (6MST or 6MWT) was performed.

This study was not blinded due to the number of assessors and nature of the interventions evaluated, which made it impossible to conceal the experimental condition from the participants or assessors. For example, participants needed to be aware of the type of exercise they received. The test order was randomized to minimize potential biases and strengthen the validity of the results. Randomization was performed *via* a draw, whereby the name of each test was written on separate pieces of paper, which were then folded and placed into an opaque container. Researchers drew a piece of paper to determine which test would be conducted on the first day of assessment and which would be scheduled for the second day, ensuring random and unbiased allocation.

All tests were conducted at the same time of day (afternoon) in an indoor environment to minimize temperature variations and other interferences. In addition, the assessors were trained by an experienced professional to carry out the pulmonary function test and administer the assessments.

Anthropometric assessment: Body weight was measured using a bioimpedance scale (AVANUTRI®; AVA-450, Rio de Janeiro, Brazil), which was adjusted according to the patient’s parameters, while height was measured with a stadiometer (Welmy®; Santa Bárbara d’Oeste, Brazil). The individuals were instructed to remove their shoes and step on the scale, placing their feet in the designated area. Subsequently, still barefoot, they were positioned for height measurement. After obtaining anthropometric values (body weight and height), the body mass index (BMI) was calculated using the formula body weight/height^2^ (kg/m^2^). Patients were classified based on their BMI as underweight (<18.5 kg/m^2^), normal weight (18.5–24.99 kg/m^2^), overweight (25–29.99 kg/m^2^), and obese (>30 kg/m^2^) (WHO, 2025).

Functional capacity assessment: The 6MST and 6MWT were conducted. In the 6MST, the individual was instructed to step up and down on a 20 cm step as many times as possible in 6 min. The step was positioned on a stable surface to prevent falls and patient discomfort during testing. Use of the upper limbs for support was not allowed during the test. The highest number of steps completed was recorded for the study. Reduced exercise tolerance was defined as performing 50% or less of the predicted value, calculated using the formula proposed by Arcuri et al. (2016). The 6MWT was performed in a 30-m flat corridor, with the patient instructed to walk as far as possible in 6 min (Holland et al., 2014; Singh et al., 2014).

Both tests were conducted by two assessors, one to lead and the other to count the number of steps and laps. Before starting, the assessors demonstrated the test procedure to ensure proper understanding. The 6MWT and 6MST followed the American Thoracic Society (ATS) (ATS Committee on Proficiency Standards for Clinical Pulmonary Function Laboratories, 2002) recommendations for the 6MWT, including standardized encouragement phrases every minute. Heart rate (HR), peripheral oxygen saturation (SpO_2_), dyspnea, and lower limb fatigue were also assessed (Borg, 1982). A minimum interval of 30 min between the first and second tests was established to allow cardiorespiratory parameters to return to baseline. Tests could be stopped if the patient experienced any limiting symptoms without stopping the timer.

Evaluation of lower limb perceived exertion and degree of dyspnea: this was assessed using the modified Borg Scale. The scale ranges from 0 to 10 points, with higher scores indicating greater lower limb fatigue or worse dyspnea (Arcuri et al., 2016). The scale was previously explained to the patient to ensure proper understanding and application during the procedure.

### Sample size

The sample size was estimated on a two-tailed significance level of 0.05, power of 90%, 20% dropout rate, and an intraclass correlation coefficient (ICC) of 0.90, resulting in a sample size of 30 to 34 patients. The calculation was performed using the G*Power program.

## Statistical analysis

Data were analyzed using SPSS software, version 23.0 (IBM Corporation, Armonk, NY, USA). Descriptive and inferential statistics were used to present the data, with results expressed as means and standard deviations. A significance level of 95% (*p* < 0.05) was established. The Kolmogorov-Smirnov test was used to assess data normality. In order to compare the physiological responses between the 6MST and the 6MWT, paired t-tests were used for parametric data, and the Wilcoxon test for non-parametric data.

Pearson’s correlation coefficient and its non-parametric counterpart, Spearman’s rank correlation coefficient, were used to evaluate the correlation between the 6MST and 6MWT, and between the variables analyzed. Intraobserver reliability was determined using the ICC for a two-way model with absolute agreement (two-way ICC) and a 95% confidence interval. ICC was interpreted according to the classification by Koo & Li (2016), where ICC < 0.50 indicates poor reliability; 0.50 to 0.75 moderate reliability; 0.75 to 0.90 good reliability; and ICC > 0.90 excellent reliability. Bland-Altman plots were used to visualize the agreement between the two tests. Validity was tested with the hypothesis of an ICC ≥ 0.70 between the number of steps in the 6MST and the distance covered in the 6MWT.

## Results

A total of 34 patients were assessed. Of these, two were excluded, one due to visual impairment and one to musculoskeletal deformity. The final sample consisted of 32 hemodialysis patients, with the majority being men (67%; *n* = 22). The average age of the sample was 57 ± 13 years, average body mass index (BMI) 28.6 ± 5.2 kg/m^2^, and average hemodialysis session duration 3.51 h.

With respect to pulmonary function, 19 patients had normal pulmonary function, one obstructive pulmonary function, four restrictive pulmonary function, and eight were unable to perform the three reproducible maneuvers, three due to persistent coughing during the test, one because of an open catheter dressing, and four who did not understand how to execute the maneuvers, leading to incomplete and unacceptable performance. None of the patients refused to participate or withdrew from the test. Sample characteristics are presented in Table 1.

**Table 1:** Sample characterization of the hemodialytic individuals.

Variables	*n*	Mean ± SD	
Age, years	32	57 ± 13	
BMI, kg/m^2^	32	28.6 ± 5.2	
		Absolute	% Predicted
FEV_1_ (L), %pred	24	2.62 ± 0.84	86.5 ± 15.6
FVC (L), %pred	24	3.25 ± 0.98	88.2 ± 13.8
FEV_1_/FVC (L), %pred	24	3.12 ± 0.90	96 ± 8.5

**Notes:**

Table 1 contains data on the characterization of the study participants.

SD, Standard deviation; %pred, percentage of predicted value; BMI, body mass index; L, liters; F, female; M, male, FEV_1_, forced expiratory volume in 1 s; FVC, forced vital capacity; and FEV_1_/FVC ratio expressed in absolute values and percentage.

**Figure 1:**
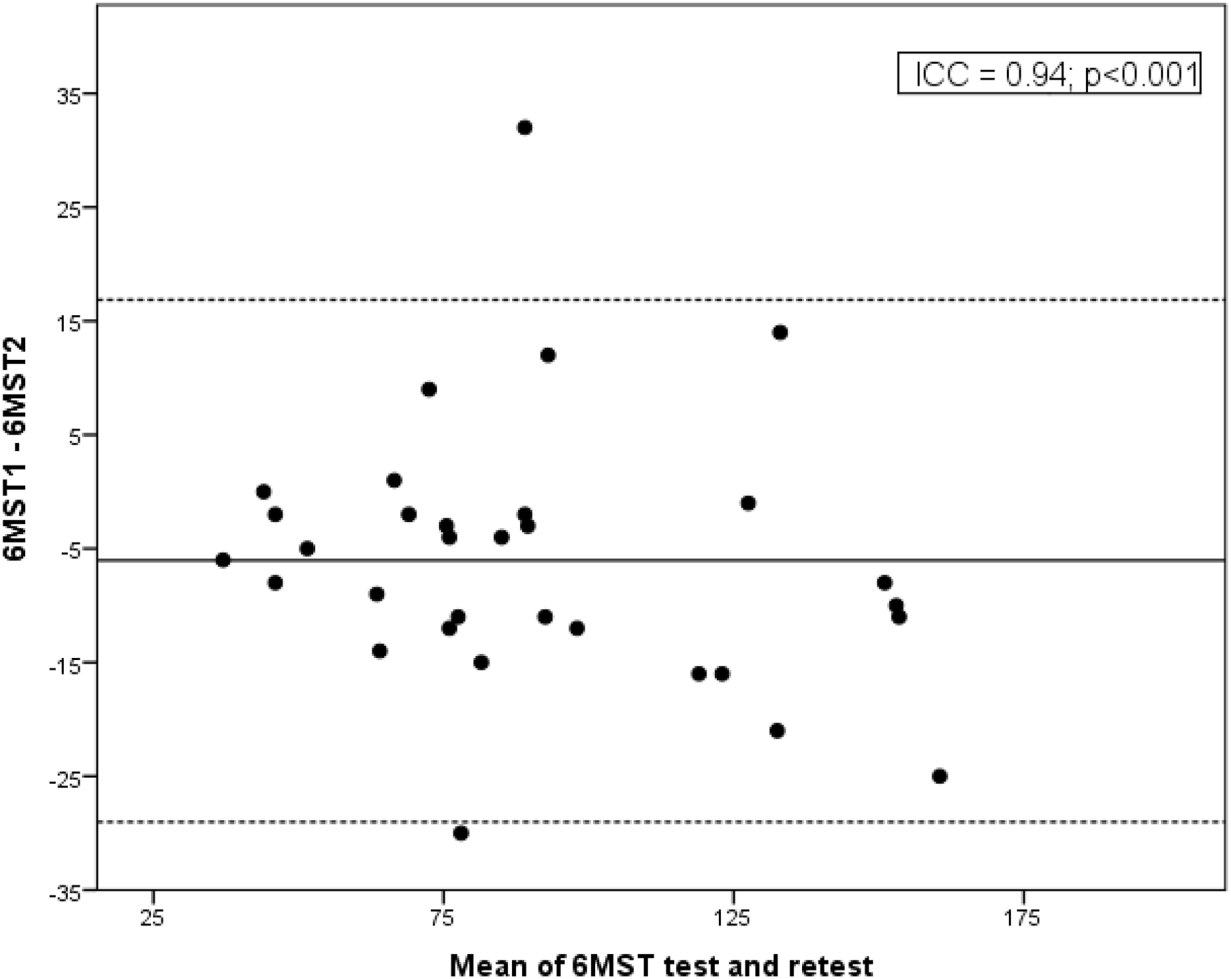
Bland-Altman plot showing the agreement between the first (6MST1) and second test (6MST2). The 6MST demonstrated high reliability, with an ICC of 0.94 (95% CI [0.85–0.97]; *p* < 0.001).

**Figure 2:**
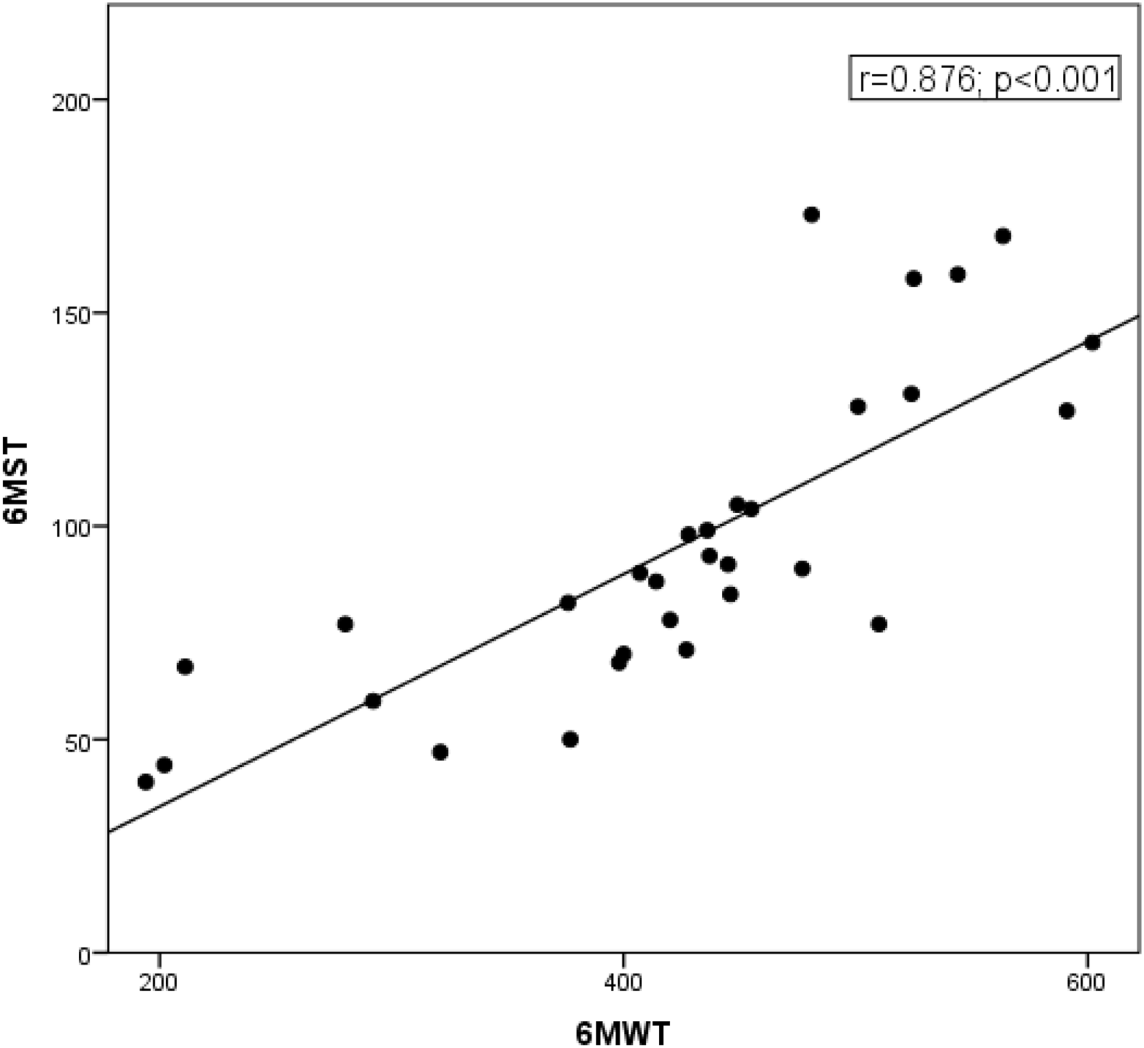
Correlation between 6MST and 6MWT. The number of steps achieved correlated with the distance covered in the 6MST (r = 0.87; *p* < 0.001).

The 6MST demonstrated high reliability, with an ICC of 0.94 (95% CI [0.85–0.97]; *p* < 0.001) (Fig. 1). The number of steps achieved correlated with the distance covered in the 6MST (r = 0.87; *p* < 0.001) (Fig. 2). The average number of steps achieved was 97 ± 38 in the 6MST, and the average distance covered in the 6MWT was 431 ± 113 m. In terms of functional capacity, 25% (*n* = 8) showed reduced performance in the 6MST (<50% of the predicted value). Additionally, the 6MST demonstrated a significant negative correlation with age (r = −0.72; *p* < 0.001), suggesting that advancing age is associated with a decreased total step count and, consequently, reduced functional capacity.

**Figure 3:**
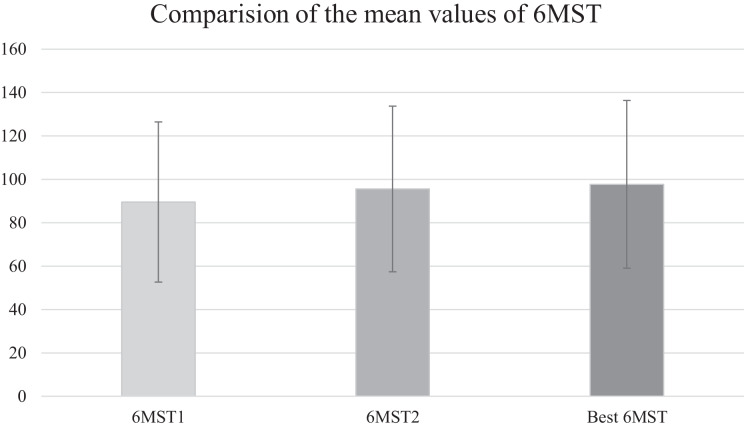
Comparision of the mean values of 6MST. The figure shows the average number of steps achieved in the first 6-min step test (6MST1), the second 6-min step test (6MST2), and the best 6-min step test.

Patients climbed more steps in the second test when compared to the first (89.5 ± 37 *vs.* 95.5 ± 38, respectively; *p* = 0.004), with an average difference of six steps. A total of 27 patients (81%) performed better in the second test, with a 7% learning effect. The data from the first and second 6MST are shown in Fig. 3.

The physiological behavior was similar between the two 6MST in all variables except for subjective perception of dyspnea and lower limb fatigue after the test, with an average increase of one point in the second 6MST (Table 2).

**Table 2:** Physiological behavior between 6MST and 6MWT.

	Mean and standard deviation		Confidence interval		*p*	Mean and standard deviation		Confidence interval		*p*
Variables	6MST1	6MST2	6MST1	6MST2		6MST	6MWT	6MST	6MWT	
Initial systolic blood pressure	140 ± 22.02	140 ± 21.21	85–180	85–180	0.418	140 ± 21.21	140 ± 20.76	85–180	100–180	0.128
Final systolic blood pressure	170 ± 32.31	180 ± 31.45	110–250	115–240	0.453	180 ± 31.45	160 ± 30.90	115–240	100–250	0.010
Initial diastolic blood pressure	80 ± 10.18	80 ± 9.52	60–100	60–100	0.768	80 ± 9.52	90 ± 10.15	60–100	60–110	0.052
Final diastolic blood pressure	80 ± 10.60	80 ± 11.35	60–110	60–110	0.272	80 ± 11.35	90 ± 12.68	60–110	70–110	0.003
Initial heart rate	82 ± 17.27	84 ± 18.34	58–120	59–131	0.307	84 ± 18.34	85 ± 16.76	59–131	62–125	0.246
Final heart rate	107 ± 26.67	112 ± 27.37	67–174	67–166	0.066	112 ± 27.37	101 ± 27.09	67–166	58–165	0.006
Initial SpO_2_	97 ± 0.93	97 ± 1.22	95–99	94–99	0.735	97 ± 1.22	97 ± 1.38	94–99	93–99	0.114
Final SpO_2_	97 ± 1.09	97 ± 1.68	95–99	92–99	0.216	97 ± 1.68	96 ± 2.46	92–99	87–99	0.020
Initial BORG dyspnea	0 ± 1.23	0 ± 1.42	0–4	0–5	0.507	0 ± 1.39	0 ± 1	0–5	0–3	0.081
Final BORG dyspnea	3 ± 2.14	4 ± 2.50	0–8	0–10	0.035*	4 ± 2.36	1 ± 2.24	0–10	0–9	<0.001*
Initial BORG LL Fatigue	0 ± 1.66	0 ± 1.60	0–6	0–6	0.188	0 ± 1.39	0 ± 1.42	0–6	0–5	<0.001*
Final BORG LL Fatigue	4 ± 2.56	3 ± 2.75	0–10	0–10	0.011*	3 ± 2.73	2 ± 2.75	0–10	0–10	<0.001*

**Note:**

The data show the physiological behavior between the two 6MSTs and between the 6MST and 6MWT. The * indicates a statistically significant difference.

Table 3 the variations in physiological parameters and subjective perception of effort during the 6MWT and 6MST. Significant differences were observed between the tests in terms of SBP, DBP, HR, and SpO2 at the end of the test. HR, SpO2, SBP, and DBP increased following the 6MST. The subjective perceived exertion at the end of the test increased by two and three points in dyspnea and lower limb weakness, respectively.

**Table 3:** Variation in physiological behavior after the 6MWT and 6MST.

	Mean and standard deviation		Min-max	
Variables	6MST	6MWT	6MST	6MWT
Δ Systolic BP	30 ± 17.3	20 ± 20.2	10–70	10–90
Δ Diastolic BP	5 ± 9.9	10 ± 11.7	20–30	30–40
Δ HR	30 ± 21.3	11 ± 16.8	28–72	9–61
**Δ** SpO_2_	0 ± 1.4	1 ± 1.75	−3 to 4	−1 to 6
**Δ** BORG Dyspnea	3 ± 1.7	1 ± 2.5	0–7	1–10
**Δ** BORG LL Fatigue	3 ± 2.7	0 ± 3.0	0–10	1–4

**Notes:**

Variations in physiological parameters and subjective perception of effort during the 6MWT and 6MST.

PA, Blood pressure; HR, heart rate; SpO2, peripheral oxygen saturation; LL, lower limbs.

## Discussion

This study demonstrated that the 6MST is reliable and valid for assessing functional capacity in hemodialysis patients, since it showed high test-retest reliability and a strong correlation with the distance covered in the 6MWT, making it suitable for clinical practice In addition, a learning effect of 7% was found, indicating the completion of two tests.

Both the 6MWT and 6MST are simple to monitor, and physical performance can be easily recorded, allowing exercise capacity to be evaluated during routine assessments (Junqué Jiménez et al., 2020). They also offer significant advantages in terms of reduced costs and increased frequency of functional assessments when compared to the CPET. Considering only applicability, both the 6MST and 6MWT could be used to assess the functional capacity of different populations due to their psychometric characteristics and lower limb (LL) use. However, the 6MST has the advantage of using a cheap and portable step ergometer, which can be transported and set up in a small room or cubicle, requiring less physical space when compared to the 30-m 6MWT circuit. Additionally, the 6MST simulates a less common and more challenging situation, namely climbing steps, for those with limitations in activities of daily living (Dal Corso et al., 2007; de Andrade et al., 2012; Lee, 2018).

The high reliability of the 6MST in this study was confirmed by its consistency during application of the two tests on the same day with a minimum interval of 30 min. The ICC value obtained was 0.94 (ICC: 0.85 to 0.97). The improved performance in the retest is likely due to familiarization with the first test. Thus, there was a 7% learning effect, indicating that the individual needs to become accustomed to the effort required, by neuromuscular adaptation to the task and a decline in possible limiting factors (Mänttäri et al., 2018). This effect is also observed in the 6MWT (Singh et al., 2014). Analysis of the learning effect is important because it demonstrates that the 6MST must be performed twice to ensure its reliability.

In the present study, the 6MST was validated using concurrent validity. Correlations between the 6MST and 6MWT were observed in both absolute values and as percentages of predicted values, considering the validity hypothesis. Performance averaged 97 ± 38 steps in the 6MST and 431 ± 113 m covered in the 6MWT. There was a strong positive correlation between the number of steps in the 6MST and the distance covered in the 6MWT (r = 0.87; *p* < 0.001), demonstrating that the 6MST is a valid test for assessing the functional capacity of hemodialysis patients.

Although the 6MST is not being widely used for individuals with CKD, likely due to a lack of documented measurement properties in the literature, it is important to underscore that the 6MST has been validated in several populations, such as those with COPD and heart failure, to assess low physical capacity (Ritt et al., 2021). Additionally, in this same population, studies have demonstrated excellent reproducibility for the 6MST, good responsiveness, as well as being a predictor for low exercise capacity and worse prognosis for these patients (Ritt et al., 2021; Costa et al., 2014; Marrara et al., 2012). Although patients with COPD and heart failure have different pathophysiological processes when compared to kidney disease, both populations experience significant exercise limitations and similar systemic alterations.

According to Costa et al. (2014), the 6MST has also been deemed valid and reliable for assessing exercise tolerance in healthy individuals, where performance in the 6MST was strongly correlated with the 6MWT. The test is widely used and studied, with well-established assessment criteria for physical capacity in other populations, making it a safe point of comparison and validation for other instruments (Costa et al., 2014). Marinho et al. (2021) demonstrated the validity and reliability of the 6MST for assessing functional capacity in individuals with advanced heart failure, using peak VO_2_ in the CPET and the number of steps in the 6MST (r = 0.71, *p* < 0.001).

By contrast, Silva et al. (2013) compared the 6MST and 6MWT in patients after a stroke and found that physiological responses were similar in both tests, but there was no correlation between the distance covered and the number of steps in the 6MST. These discrepancies, compared to the present study, may be explained by the pathophysiology of stroke being different from metabolic and cardiorespiratory diseases, given that stroke primarily affects motor function and mobility more than the cardiorespiratory system itself.

Currently, there is no established cutoff point in the literature for discriminating hemodialysis patients with impaired functional capacity, as assessed by the 6MST. In other populations, such as those with COPD, cutoff points of <78 steps on the first 6MST and <86 steps on the second have been associated with reduced exercise capacity (Pessoa et al., 2014). Ritt et al. (2021) observed that a cutoff of >105 steps is related to achieving a peak VO_2_ above 20 mL·kg^−1^·min^−1^ in individuals with heart failure.

The primary goal of the 6MST as a submaximal test is to determine cardiorespiratory fitness. Similar to the 6MWT, the 6MST is safe and can be performed at submaximal effort, albeit with slightly higher energy expenditure. da Costa et al. (2017) conducted both the 6MST and 6MWT on healthy, sedentary volunteers and in addition to confirming that the former is safe, found that it caused greater changes in HR without reaching HRmax, thereby confirming its submaximal nature.

In the present study, ventilatory and cardiovascular variables between the submaximal tests showed similar responses, demonstrating comparable demands. However, a greater ΔHR and ΔLL fatigue was observed with the 6MST compared to the 6MWT, corroborating Costa et al. (2014) This suggests that while the 6MST is a safe submaximal test, it requires slightly more from hemodialysis patients when compared to the 6MWT.

The Borg scale scores for dyspnea increased by four points at the end of the test, and lower limb fatigue increased by three points. Significant increases in ΔLL fatigue were obtained with the 6MST when compared to the 6MWT. This could be due to the higher amount of active muscle mass involved in climbing up and down steps, in addition to mechanical differences in movement, gravity effects, and postural changes. Although this difference does not appear to affect test performance, the higher ΔHR values found with the 6MST compared to the 6MWT suggest greater cardiovascular stress, likely due to peripheral metabolic demands and postural variations involved in the test. These finding indicate that it not only challenges cardiovascular endurance but also engages larger muscle groups, which can provide more detailed insights into both the ventilatory and cardiovascular responses to physical stress.

The 6MST can be used to assess the functional capacity of CKD patients and improve patient outcomes by identifying those at risk, which influences decision-making regarding treatment. It should be implemented in accordance with ATS recommendations (ATS Committee on Proficiency Standards for Clinical Pulmonary Function Laboratories, 2002) and is more valuable when combined with additional assessments, such as tests for strength, endurance, and quality of life, providing a more comprehensive assessment of patients’ overall condition. Although it lacks further psychometric data, it can still be used for pre and post-physiotherapy comparisons.

Potential challenges when implementing the 6MST include the fact that the test might be more demanding for some patients because it causes greater fatigue than the 6MWT. This is supported by the physiological responses observed in the presente study and the fact that climbing up and down stairs is less common in everyday life than walking, as performed in the 6MWT. Additionally, CKD patients often have different comorbidities, which may affect their ability to perform the test. For example, patients with orthopedic limitations may be unable to perform the test due to the need for hip flexion or complete it in a way that does not accurately reflect their true functional capacity. Moreover, patients who are severely debilitated may not tolerate the test due to fatigue.

This is the first study investigating the use of the 6MST in hemodialysis patients, demonstrating its reliability and validity. As such, it provides a new potential use for this tool in assessing and managing the functional status of these patients. These results have potential implications for nephrology and chronic disease research by encouraging further investigations in similar patient populations.

### Study limitations

This was a single-center study with a relatively small sample, which may limit the generalizability of the findings. Additionally, factors such as BMI, age, and comorbidities vary in patients undergoing hemodialysis, which may have influenced the results and should be investigated in future research. The study could be complemented by a longitudinal follow-up to examine the stability of the 6MST over time. Incorporating follow-up assessments and a control-group into future investigations could provide more comprehensive insights into the test’s reliability and ability to assess functional capacity in chronic kidney disease patients over time. Furthermore, it is worth noting that, due to the small sample size and the aim of generalizing the results, outliers were included in the analysis. Further research is needed to explore other 6MST measurement properties and establish it as a robust functional assessment tool for patients with chronic kidney disease.

## Conclusion

The 6MST provides a reliable and valid measure of functional capacity in hemodialysis patients, suitable for routine clinical assessments. Due to the learning effect observed in this population, it is recommended to perform two tests on the same day 30 min apart. The 6MST is effective in hemodialysis patients who exhibit systemic changes and reduced exercise tolerance. It allows for constant monitoring of cardiorespiratory variables and can be incorporated into the daily routine of clinics and healthcare services. These findings could contribute to better patient assessment, management, and decision-making, improving patient outcomes.

Additional studies on the long-term stability, responsiveness, and impact of the 6MST on patient-centered outcomes are recommended to improve understanding of its clinical usefulness. Moreover, further research with different hemodialysis populations, including those with varying comorbidities or in different clinical settings, is essential to improve the test’s applicability and relevance in broader clinical practice.

## Supplemental Information

10.7717/peerj.19157/supp-1 Supplemental Information 1 Raw data. Sex, age, height, weight, 16MST, 26MST, best 6MST, and best 6MWT. These data were used for the main statistics of the study. 6MST—six-minute step test. 6MWT—six-minute walk test.

## Additional Information and Declarations

### Data Availability

The following information was supplied regarding data availability:

The raw measurements are available in the Supplemental Files.
